# CRISPR/Cas9 mediated genome editing of *Caenorhabditis nigoni* using the conserved *dpy-10* co-conversion marker

**DOI:** 10.17912/micropub.biology.000937

**Published:** 2023-08-30

**Authors:** Charlotte P. Choi, Anne M. Villeneuve

**Affiliations:** 1 Department of Developmental Biology, Stanford University School of Medicine, Stanford, CA 94305 U.S.A.

## Abstract

In this study, we developed an efficient co-conversion marker, using the conserved *dpy-10* gene, to facilitate creation and detection of CRISPR/Cas9-mediated targeted genomic changes in an emerging male/female nematode model system, *Caenorhabditis nigoni* .

**Figure 1. Alignment of partial DPY-10 amino acid sequences from C. elegans and C. nigoni:**
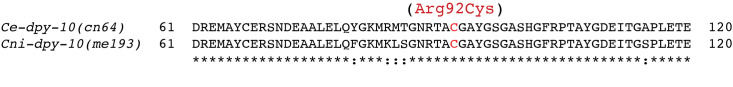
The JU1422 reference genome was used to derive the amino acid sequence of Cni-DPY-10. Red colored Cysteine depicts Arg92Cys amino acid substitution that causes a dominant Rol phenotype in both *C. elegans* and *C. nigoni* .

## Description

CRISPR/Cas9-mediated genome engineering has become a powerful and efficient strategy for creating targeted mutations in the *Caenorhabditis elegans* model system. In the *C. elegans* system, use of dominant co-conversion markers has proven to be an effective approach for identifying worms likely to carry a new edit in a gene of interest (Arribere et al. 2014) . Here, we sought to adapt the efficient genome editing strategies developed for *C. elegans* for use in *Caenorhabditis nigoni* , a male/female species that is an emerging alternative nematode model system (Woodruff et al. 2010; Yin et al. 2018; Yu et al. 2015) . Specifically, we developed a co-conversion marker utilizing the conserved *dpy-10* gene.

Because the *C. elegans* and *C. nigoni* DPY-10 proteins are 98% identical, we reasoned that we could likely recapitulate the gain-of-function Rol phenotype caused by the *C. elegans* *dpy-10 ( cn64 )* mutation by using CRISPR/Cas9 editing to introduce a missense mutation causing the identical Arg92Cys (R92C) amino acid substitution into the *C. nigoni* *dpy-10* coding region (Arribere et al. 2014; Paix et al. 2015; Figure 1 ). We created this mutation using a ssDNA repair template harboring the corresponding mutation in the *C. nigoni* *dpy-10* sequence, and we confirmed that heterozygosity for this new allele, *Cni* - *dpy-10 (me193),* does indeed cause the same distinct left rolling phenotype for both *C. nigoni* males and females as observed in *C. elegans* worms heterozygous for *Ce-dpy-10 ( cn64 )* .

Moreover, we have demonstrated that *Cni* - *dpy-10 (R92C)* can serve as an efficient co-CRISPR repair marker for introducing other edits into the *C. nigoni* genome. We injected Cas9 complexes loaded with *Cni* - *dpy-10* and *Cni* - *htp-3* sgRNAs together with *Cni* - *dpy-10 (R92C)* template DNA and with *Cni-htp-3::3xFlag* template DNA designed to introduce a 3xFlag tag sequence at the C-terminus of HTP-3 . 8/14 injected animals produced Rol progeny indicative of heterozygosity for the *Cni* - *dpy-10 (R92C)* mutation; we also observed a few rare Dpy progeny, which are likely indicative of other allelic pair combinations at the *Cni-dpy-10* locus ( *i.e.* *R92C/null* , *R92C/R92C* and/or *null/null* ).

Unlike *C. elegans* , *C. nigoni* worms are not self-fertile. Thus, to ensure that F1 progeny were produced from injected *C. nigoni* females, young adult females were allowed to mate with several males for 12 hours prior to injection and again with two to three males for 2-3 hours post-injection. From each successful injectant, 32 L4 stage F1 Rol females were singled out, and each was allowed to mate with 2-3 wild-type males to produce F2 progeny. These F2s were a mixture of Rol and non-Rol worms, consistent with F1 Rol worms being heterozygous for a *Cni* - *dpy-10 (R92C)* mutation. Once viable F2s were laid, we performed PCR to genotype each of the 32 singled F1s worms using primers amplifying the C-terminal region of the *htp-3* locus. 59% of the F1 Rol worms were heterozygous for the templated 3xFlag-encoding insertion at the *htp-3* locus, and none of the F1 Rol worms were homozygous for the 3xFlag insertion. Finally, to generate lines homozygous for the *Cni-htp-3::3xFlag* edit, we set up one-to one-matings between non-Rol males and non-Rol virgin females descended from an F1 that had segregated a sequence-verified *Cni-htp-3::3xFlag* edit, as indicated in the Methods. We caution researchers accustomed to working with self-fertilizing hermaphroditic Caenorhabditis species that it is important to derive multiple separate lines homozygous for a desired edit, as dioecious *Caenorhabditis* nematodes have been shown to exhibit significant inbreeding depression that can lead to reduced fecundity and eventual extinction of inbred lines (Dolgin et al. 2007) .

Together, our data indicate that successful and efficient editing of the *C. nigoni* genome can be achieved using a powerful co-CRISPR approach developed for *C. elegans* . Our method and the approach described in the accompanying Micropublication by Harbin and Ellis (2023) will facilitate sophisticated genetic analyses using the *C. nigoni* system.

## Methods

*Injection of pre-formed Cas9 RNP complexes* :

The concentrations of components in the CRISPR-Cas9 injection mix, adapted from the protocol described in Paix et al. 2015, are described in table below.

Injection Mix:

**Table:** 

Name/Description	Concentration	Volume
*Cni-dpy-10* crRNA	8 μg/μL	0.675 μL
*Cni-htp-3* crRNA	8 μg/μL	0.5 μL
tracrRNA	4 µg/µL	2.5 µL
Cas9 protein	10 µg/µL	2.5 µL
Repair template for *Cni-htp-3-3xFlag*	1 µg/µL	1.5 µL
Repair template for *Cni-dpy-10 (R92C) *	1 µg/µL	0.5 µL
Resuspension buffer	--	1.825 µL

*Generating and verifying lines homozygous for desired edit:*

Following one-to-one matings between descendants of F1s that had segregated a sequence-verified edit in a gene of interest (in this case *Cni-htp-3::3xFlag* ), progeny resulting from these pairwise mating events are left to propagate for 3-4 days; from each individual mating plate, multiple worms are pooled together for PCR-based genotyping. It is recommended to set up at least 48 pairwise matings based on a 1/16 probability that both the female and the male in a mating pair will be homozygous for the desired edit. However, it is advisable to set up even more matings, both to account for mating inefficiency and to ensure isolation of multiple prospective homozygote lines as a hedge against negative impacts of inbreeding. Once plates have been identified that appear to segregate only the desired edited allele, one-to-one pairwise matings should be repeated using worms derived from such plates, followed by genotyping of the individual worms in the mating pair and/or their progeny, to confirm that a strain homozygous for the desired edit has been established.

## Reagents

All sgRNA, tracrRNA, ssDNA repair templates were ordered from IDT and Cas9 protein was ordered from PNA Bio. Sequences used for template ssDNAs and sgRNAs were derived from the *C. nigoni* genome assembly with GenBank accession PDUG00000000 retrieved from Wormbase ParaSite Version: WBPS18 (WS285) (Howe et al. 2016) .

Guide sequence used to target the *Cni-dpy-10* locus (Genome assembly: PRJNA384657, Scaffold: CM008510.1, Location: 6,129,503-6,130,717): CATAAGCACCACGAGCGGTA

Guide sequence used to target the sequence coding for the C-terminus of Cni-HTP-3 (Genome assembly: PRJNA384657, Scaffold: CM008510.1, Location: 4,616,267-4,617,819): TGAGACAATAATAGTTATTT

*Cni-dpy-10 (R92C)* Repair template sequence: CTCTAGAACTTCAATTCGGCAAAATGAAACTCTCCGGAAACCGTACCGCTTGTGGTGCTTATGGAAGTGGAGCTTCTCATGGATTCAGACCAACTGCTTATG

*Cni-htp-3::3xFlag* Repair template sequence: TCAAGTACAGCCAATCCGCTTCTCTTCGACCAAAAGGAGGCTCAGGAATGGGATCGGACTATAAAGATCACGACGGAGATTACAAGGACCATGATATCGACTACAAGGACGACGACGACAAGGGATAACTATTATTGTCTCAGGTTATCATGTGCTATCT

Forward primer for *Cni-dpy-10* sequencing: AACTATTCGCGTCAGATGACGTA

Reverse primer for *Cni-dpy-10* sequencing: TTGAGTGGACAGGTCTGATTTGG

Forward primer for *Cni-htp-3* sequencing: TCAAAGCTTTACTATATGCATGCATAACTAA

Reverse primer for *Cni-htp-3* sequencing: CATGTCCAGCAACCAGCAG

*C. nigoni* strain used for injections: JU1422 .
